# Human Adipose Tissue Derived Mesenchymal Stem Cells Aggravate Chronic Cyclosporin Nephrotoxicity by the Induction of Oxidative Stress

**DOI:** 10.1371/journal.pone.0059693

**Published:** 2013-03-26

**Authors:** Byung Ha Chung, Sun Woo Lim, Kyoung Chan Doh, Shang Guo Piao, Seong Beom Heo, Chul Woo Yang

**Affiliations:** 1 Convergent Research Consortium for Immunologic Disease, Seoul St. Mary’s Hospital, College of Medicine, The Catholic University of Korea, Seoul, Korea; 2 Transplant Research Center, Seoul St. Mary’s Hospital, College of Medicine, The Catholic University of Korea, Seoul, Korea; 3 Division of Nephrology, Department of Internal Medicine, Seoul St. Mary’s Hospital, College of Medicine, The Catholic University of Korea, Seoul, Korea; Institut national de la santé et de la recherche médicale (INSERM), France

## Abstract

The aim of this study was to investigate whether hATMSCs protect against cyclosporine (CsA)-induced renal injury. CsA (7.5 mg/kg) and hATMSCs (3×10^6^/5 mL) were administered alone and together to rats for 4 weeks. The effect of hATMSCs on CsA-induced renal injury was evaluated by assessing renal function, interstitial fibrosis, infiltration of inflammatory cells, and apoptotic cell death. Four weeks of CsA-treatment produced typical chronic CsA-nephropathy. Combined treatment with CsA and hATMSCs did not prevent these effects and showed a trend toward further renal deterioration. To evaluate why hATMSCs aggravated CsA-induced renal injury, we measured oxidative stress, a major mechanism of CsA-induced renal injury. Both urine and serum 8-hydroxydeoxyguanosine(8-OHdG) levels were higher in the CsA+hATMSCs group than in the CsA group (*P*<0.05). An in vitro study showed similar results. Although the rate of apoptosis did not differ significantly between HK-2 cells cultured in hATMSCs-conditioned medium and those cultured in DMEM, addition of CsA resulted in greater apoptosis in HK-2 cells cultured in hATMSCs-conditioned medium. Addition of CsA increased oxidative stress in the hATMSCs-conditioned medium. The results of our study suggest that treatment with hATMSCs may aggravate CsA-induced renal injury because hATMSCs cause oxidative stress in the presence of CsA.

## Introduction

Cyclosporin A (CsA) is used widely after organ transplantation and to treat various disorders such as autoimmune diseases and primary glomerulonephritis. However, despite the clinical efficacy of CsA, nephrotoxicity is considered a major dose-limiting adverse effect [1]. Long-term administration of CsA causes progressive renal failure and irreversible renal striped interstitial fibrosis, inflammatory cell infiltration, and hyalinosis of the afferent glomerular arterioles [2]. Long-standing hypoxic and ischemic injury caused by vasoconstriction is regarded as the main etiology of CsA-induced injury through an increase in the production of reactive oxygen species, which cause cellular injury and promote apoptotic cell death [3]. Direct activation of apoptosis genes by CsA and inflammatory cell infiltration by activation of the innate immune system have also been proposed as important mechanisms [4], [5].

Mesenchymal stem cells (MSCs) are of interest because of their potential therapeutic effects in various disorders [6]. This therapeutic potential is mediated by multiple mechanisms such as immunomodulatory effects through the secretion of regulatory cytokines, activation of regulatory immune cells, and the capacity to increase cellular repair through the secretion of antiapoptotic, antifibrotic, and proangiogenic factors [7], [8], [9]. These multiple functions of MSCs may lead to multifaceted strategies in various organs and diseases. The usefulness of MSCs in treating kidney disorders has been investigated extensively in acute and chronic kidney disease models, and the results are promising [10], [11], [12], [13].

Considering the known therapeutic effects of MSCs, we postulated that MSCs may have a therapeutic effect on CsA-induced nephrotoxicity. In this study, we used human adipose tissue-derived mesenchymal stem cells (hATMSCs) and our established rat model of chronic CsA-induced nephrotoxicity to assess the effect of MSCs on CsA-induced nephrotoxicity. We focused mainly on two aspects: whether hATMSCs are a therapeutic option in the treatment of CsA-induced nephrotoxicity and whether CsA has detrimental effects on hATMSC functions.

## Materials and Methods

### Animals and Drugs

Male Sprague Dawley rats (Charles River Technology, Seoul, Korea), weighing initially 230 to 250 g, were housed in cages (Nalge Co., Rochester, NY) in a controlled-temperature and controlled-light environment, and allowed free access to a low-salt diet (0.05% sodium, Teklad Premier, Madison, WI) throughout the experimental period. CsA (Novartis, Basel, Switzerland) was diluted in olive oil (Sigma, St Louis, MO) to a final concentration of 7.5 mg/mL.

### Isolation and Culture of hATMSCs

The MSCs used in this study were derived from surplus fat tissues obtained for a stem cell banking service and no longer needed for that purpose. Written informed consent for research use was given by the donors. In brief, human abdominal subcutaneous fat tissues were obtained by simple liposuction. hATMSCs were isolated from the fat stromal vascular fraction by their adherence to plastic and were culture-expanded as described previously [14]. Cryopreserved stem cells were stored in liquid nitrogen vapor and were thawed and recultured in growth medium (RKCM; RNL Bio, Seoul, Korea) based on the injection schedule under Good Manufacturing Practice conditions (RNL Bio, Seoul, Korea). Harvested cells were counted and tested for cell viability, purity (CD31, CD34, CD45), identity (CD73, CD90), sterility, and endotoxin and mycoplasma contamination before use.

### Experimental Design

#### Experimental protocol

**Figure 1:**
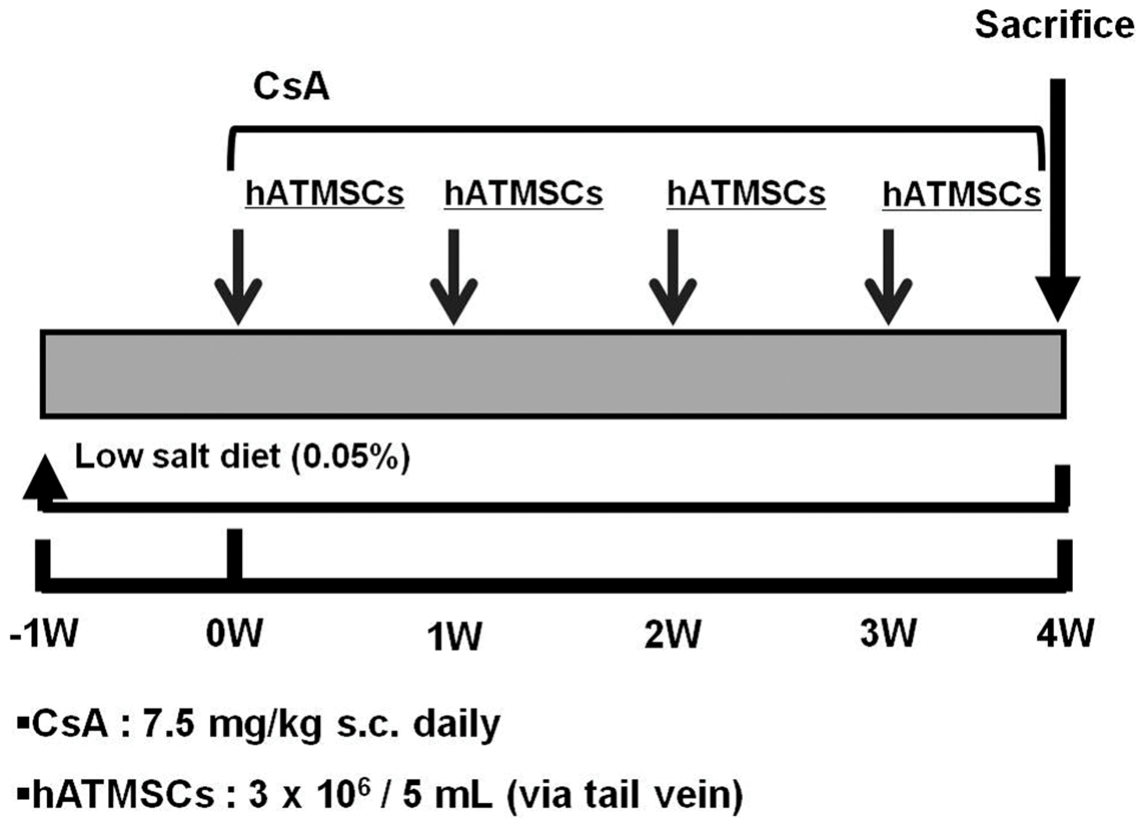
Experimental design in this study. CsA was administered at a dose of 7.5 mg/kg subcutaneously daily. hATMSCs was infused at 0,1,2 and 3 weeks from the start of CsA with cell number of 3×10^6^/mL via tail vein. hATMSCs; human adipose tissue derived mesenchymal stem cells.

The experimental protocol was approved by the Animal Care Committee of the Catholic University of Korea, and all procedures performed in this study followed ethical guidelines for animal studies. Figure 1 shows the experimental protocol of this study. Rats were randomized into the four groups listed below and were treated for 4 weeks. We subcutaneously (s.c.) administered 7.5 mg/kg CsA in olive oil or olive oil alone daily for 4 weeks. hATMSCs were injected via the tail vein at a concentration of 3×10^6^/5 mL 0, 1, 2, and 3 weeks after the start of CsA administration. The groups were as follows.

1. Vehicle (VH) group (n = 8) in which rats received olive oil (1 mL/kg per day s.c.)
2. VH+hATMSC group (n = 8) in which rats received VH and hATMSCs (3×10^6^/5 mL)
3. CsA group (n = 8) in which rats received CsA (7.5 mg/kg per day s.c.)
4. CsA+hATMSC group (n = 8) in which rats received both CsA and hATMSCs (3×10^6^/5 mL)

#### Basic protocol

After starting the treatment, rats were pair fed, and body weight (BW) was measured daily. Before sacrifice, animals were housed individually in metabolic cages (Tecniplast Gazzada S.a.r.l., Italy) for 24-h urine collection. The following day, animals were anesthetized with ketamine, and a blood sample and tissue specimens were obtained.

### Homing of MSCs

Cell homing to the injured kidney was studied using hATMSCs labeled with PKH26 fluorescent dye (Sigma) according to the technique described previously [15]. CsA (n = 2) or olive oil (n = 2) was given for 4 weeks, and PKH26-labeled hATMSCs (3×10^6^/5 mL) were infused into the tail vein. Animals were sacrificed after 24 h. Frozen kidneys were cut into 5-µm cryostat sections and air dried, and fixed in acetone for 5 min. Sections were counterstained with 4′,6-Diamidino-2-Phenylindole (Sigma) and covered with fluorescent mounting medium (DakoCytomation, Glostrup, Denmark). Preservation of tissue integrity was assessed by staining with Mayer’s hematoxylin and eosin, 1% aqueous solution (Bio-Optica, Milan, Italy). Specific fluorescence versus renal background was analyzed using a Leica DML microscope (excitation 490 nm/emission 570 nm) under 40× magnification.

### Measurement of Renal Function and Whole-blood CsA Level

Serum and urine creatinine levels were measured by an enzymatic method using a Daiichi kit (Daiichi Pure Chemical Co. Ltd, Tokyo, Japan) on a Hitachi 7600 chemistry analyzer (Hitachi Inc., Tokyo, Japan). Creatinine clearance was calculated using the standard formula (Creatinine Clearance = Urine Creatinine × Urine Volume/Plasma Creatinine). The whole-blood CsA level was measured using a monoclonal radioimmunoassay (Incstar Co., Stillwater, MN).

### Measurement of CsA-induced Interstitial Fibrosis

Kidney tissues were fixed in periodate–lysine–paraformaldehyde solution and embedded in wax. After dewaxing, 4-µm sections were processed and stained with Masson’s trichrome and hematoxylin. A finding of tubulointerstitial fibrosis (TIF) was defined as a matrix-rich expansion of the interstitium with tubular dilatation, tubular atrophy, tubular cast formation, sloughing of tubular epithelial cells, or thickening of the tubular basement membrane. A minimum of 20 fields per section were assessed using a color image analyzer (TDI Scope Eye™ Version 3.0 for Windows, Olympus, Japan). Briefly, the image was captured, and the extent of TIF was quantified using the Polygon program by counting the percentage of areas injured per field of cortex under 100× magnification, as described previously [16]. Histopathological analysis was performed in randomly selected cortical fields of sections by a pathologist blinded to the identity of the treatment groups.

### In situ TdT-mediated dUTP-biotin Nick End-labeling (TUNEL) Assay

Cells undergoing apoptosis were identified using an ApopTag in situ apoptosis detection kit (Chemicon, Temecula, CA). After dewaxing, the sections were treated with proteinase K, incubated with equilibration buffer in a humidified chamber for 10 min at room temperature, and then incubated with working-strength TdT enzyme solution in a humidified chamber at 37°C for 2 h. The reaction was terminated by incubation in working-strength stop/wash buffer for 30 min at 37°C. The sections were rinsed with phosphate-buffered saline (PBS) and then incubated with anti-digoxigenin peroxidase in a humidified chamber for 30 min at room temperature. The sections were incubated with diaminobenzidine and 0.01% H_2_O_2_ for 5 min at room temperature, rinsed with PBS, counterstained with hematoxylin, and examined using light microscopy. As a positive control, slides were treated with DNase (20 Kunitz units/mL; Sigma), and the slides for the negative control were treated with buffer lacking TdT. The numbers of TUNEL-positive cells were counted in selected areas (0.5 mm^2^) in each section under 200× magnification.

### Immunohistochemistry for ED1-positive Cells

The dewaxed sections were incubated with 0.5% Triton X-100/PBS solution for 30 min and washed with PBS three times. Nonspecific binding sites were blocked with normal horse serum diluted 1∶10 in 0.3% bovine serum albumin for 30–60 min and then incubated for 2 h at 4°C in antiserum against ED1 (Serotec, Oxford, UK) diluted 1∶1000 in a humid environment. The sections were rinsed in PBS and incubated in peroxidase-conjugated rabbit anti-mouse IgG (Amersham Pharmacia Biotech, Piscataway, NJ) for 30 min. For coloration, sections were incubated with a mixture of 0.05% 3,3′-diaminobenzidine containing 0.01% H_2_O_2_ at room temperature until a brown color was visible, washed with PBS, counterstained with hematoxylin, and examined under light microscopy. The numbers of ED1-positive cells were counted automatically as described in the apoptosis section below.

### Immunoblot Analysis for Caspase 3

For immunoblot analysis, kidney tissue was homogenized in lysis buffer (20 mM Tris-CL [pH 7.6], 150 mM NaCl, 1% [w/v] sodium deoxycholate, 1% [v/v] Triton X-100, 0.1% SDS, 2 mM NaVO_3_, and freshly added 1% [v/v] aprotinin, leupeptin [1 µg/mL], pepstatin [1 µg/mL], and 1 mM phenylmethylsulfonyl fluoride). Homogenates were centrifuged at 3000 rpm for 15 min at 4°C, and the protein concentration of the lysate was determined using the Bradford microassay method (Bio-Rad, Hercules, CA). Protein samples were resolved on 15% SDS-polyacrylamide gel electrophoresis and then electroblotted onto a Bio-Blot nitrocellulose membrane (Bio-Rad). An equal amount of protein loading (80 µg) was verified by Ponceau S staining. The membrane was blocked for 1 h in Tris-buffered saline with Tween-20 (TBS-T; 10 mM Tri-CL, 150 mM NaCl [pH 8.0], 0.05% Tween-20) containing 5% nonfat powdered milk. Caspase 3 was detected by incubating for 1 h with specific antibody (Chemicon) diluted 1∶200. The blot was incubated in the primary antibody, washed six times with TBS-T, and then incubated with secondary antibody (Amersham Biosciences, UK) conjugate at 1∶1000 for 1 h. Antibody-reactive protein was detected using enhanced chemiluminescence (ECL; Amersham Biosciences). Optical densities were obtained using the VH group as the 100% reference.

### Measurement of Urinary and Serum 8-hydroxydeoxyguanosine (8-OHdG) Levels

Twenty-four-hour urinary concentration of 8-OHdG and serum 8-OHdG were measured using a competitive ELISA (Institute for the Control of Aging, Shizuoka, Japan).

### Cell Culture and Administration of CsA and hATMSCs to HK-2 Cells

To observe tubular cell injury directly in cells treated with CsA and hATMSCs, we measured Annexin V positivity in HK-2 cells. HK-2 cells were purchased from the American Type Culture Collection (ATCC, Manassas, VA). The cells were cultured in DMEM or hATMSC-conditioned medium. Another set of HK-2 cells which were also cultured in DMEM or hATMSC-conditioned medium were treated with CsA (12.5 µM). Cells were washed twice with PBS at 4°C and resuspended in 250 µL of a combination buffer solution, and the cell concentration was adjusted to 1×10^5^/mL. Annexin V/APC (5 µL) and propidium iodide (5 µL) were added, and the cells were incubated in the dark for 15 min and then analyzed using a FACS LSRFortessa flow cytometer (BD Biosciences, San Jose, CA). We also measured oxidative stress markers in another set of cultured cells, as described below.

### Oxidative Stress Markers in hATMSC-conditioned Media with and without CsA

To investigate whether the production of oxidative stress markers by hATMSCs increased in cells incubated with CsA, we measured 8-OHdG and nitric oxide (NO-) levels in the hATMSC-conditioned medium. After 24 h of culture with or without CsA (100 µM), the hATMSC-conditioned medium was collected, and 8-OHdG and nitric oxide levels were measured using a competitive ELISA (Institute for the Control of Aging) and Griess reagent system (Promega, Madison, WI) respectively.

### Statistical Analysis

The data are expressed as mean ± SEM. Multiple comparisons between groups were performed by one-way ANOVA with the post hoc Bonferroni test using SPSS software (version 9.0). Significance was assumed at P<0.05.

## Results

### Phenotypic Analysis of hATMSCs

**Figure 2:**
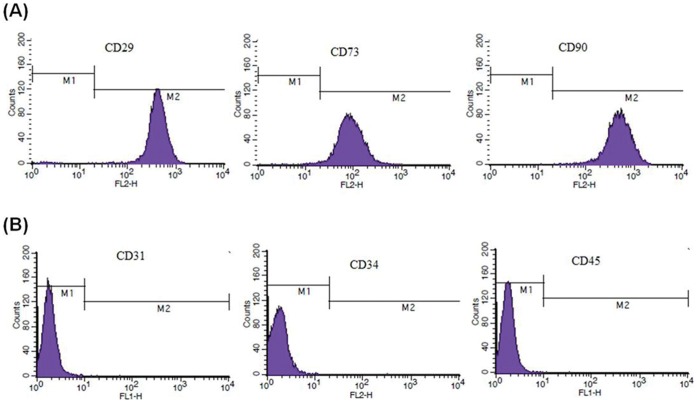
Flowcytometric analysis of surface markers on cultured hATMSCs. (**A**) MSC-specific markers CD29, CD73, CD90 and CD105 were expressed on culture hATMSCs. (**B**) Hematopoietic stem cell markers CD31, CD34 and CD45 were not expressed.

Flowcytometric analysis was performed to confirm the identity of the cells as hATMSCs. Cultured hATMSCs expressed high levels of the MSC-specific markers CD29, CD73, CD90 and CD105 (Figure 2A) but did not express the hematopoietic markers CD31, CD34 and CD45. (Figure 2B).

### In vivo Tracking of hATMSCs in the CsA and VH Groups

**Figure 3:**
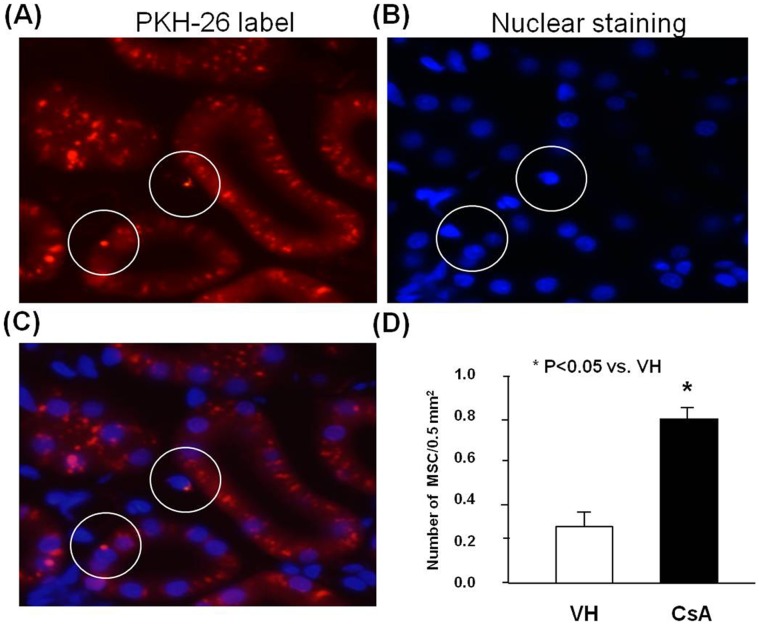
Homing of PKH-26 labeled hATMSCs to the kidney tissue. (**A**) The representative of PKH-26 label (**B**) The staining for nuclei using (DAPI) (**C**) The overlay of (A) and (B) showed homing of PKH-26 labeled hATMSCs in renal interstitial tissue. PKH-26 staining merged with nuclear staining (in the circle), indicating PKH-26 labeled hATMSCs. (Original magnification x400 in A through C) (**D**) The quantization of PKH-26 labeled hATMSCs. Labeled cell was more frequently detected in CsA treated group compared to VH group. hATMSCs; human adipose tissue derived mesenchymal stem cells DAPI; 4′,6-Diamidino-2-Phenylindole.

Homing of hATMSCs was evaluated 24 h after injection into the tail vein in rats treated with CsA (n = 2) or olive oil (n = 2) for 4 weeks. In renal tissue, PKH-26 labeled hATMSCs were found mainly in the tubule–interstitial area. Significantly more PKH-26 labeled hATMSCs were detected in the CsA group than in the VH group (0.8±0.1/0.5 mm^2^ vs. 0.26±0.09/0.5 mm^2^; P<0.05) (Figure 3).

### Effect of Treatment with hATMSCs on CsA-induced Renal Dysfunction

To evaluate the renal function of rats, serum creatinine concentration and creatinine clearance were measured at 4 weeks. Table 1 shows the baseline parameters in the experimental groups. Body weight, serum creatinine level, creatinine clearance, and CsA concentration did not differ between the groups.

**Table 1:** The effects of treatment with hATMSC on basic parameters in experimental groups.

	VH	VH+hATMSCs	CsA	CsA+hATMSCs
**ΔBody weight** **(g)**	11.8±9.1	15.7±14.3	7.3±10.7	5.6±8.7
**Scr (mg/dl)**	0.55±0.08	0.54±0.05	0.58±0.08	0.60±0.01
**ClCr (ml/min/100** **g)**	0.53±0.04	0.55±0.03	0.43±0.09	0.41±0.07
**CsA level** **(ng/mL)**	–	–	2367±86	2526±231

ΔBody weight, changes in body weight; Scr, serum creatinine; ClCr, creatinine clearance; CsA, cyclosporine, hATMSC; human adipose tissue derived mesenchymal stem cell.

* P<0.05 vs. VH group.

** P<0.05 vs. CsA group.

### Effects of Treatment with hATMSCs on Interstitial Inflammation in Chronic CsA Nephropathy

**Figure 4:**
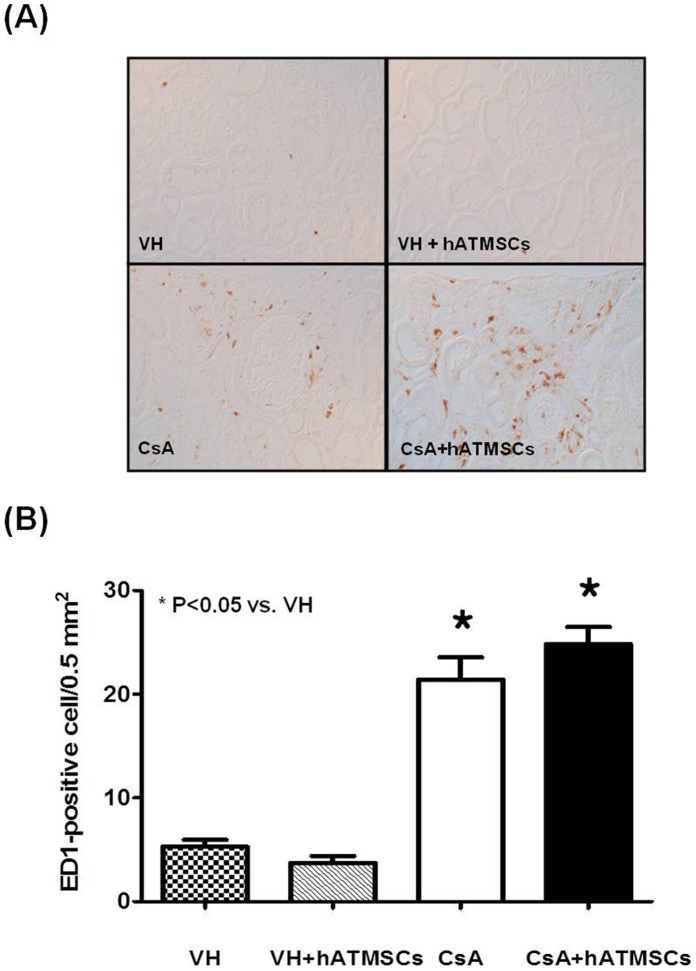
Effect of hATMSCs infusion on inflammatory cell infiltration in rats with chronic CsA nephropathy. (**A**) Representative photomicrographs of the immunohistochemical detection of ED1 protein (Original magnification x200). (**B**) Quantitative analysis of ED1-positive cells in the four groups. CsA administration increased ED1-positive cell number compared to VH and treatment with hATMSCs did not decrease it. hATMSCs; human adipose tissue derived mesenchymal stem cells.

To evaluate the effects of hATMSCs on interstitial inflammation induced by CsA administration, ED1-positive cells were detected by immunohistochemistry, and the numbers of ED1-positive cells in the experimental groups were quantified (Figure 4). ED1-positive cells were minimal in the VH group (5.3±1.8/0.5 mm^2^) and VH+hATMSC group (3.7±1.8/0.5 mm^2^). The number of ED1-positive cells was significantly higher in renal tissue from the CsA group (21.4±6.1/0.5 mm^2^) than in tissue from the VH group (P<0.05). The number of ED1-positive cells was significantly higher in the CsA+hATMSC group (24.8±4.4/0.5 mm^2^) than in the VH group but did not differ significantly from that in the CsA group (P>0.05).

### Effects of Treatment with hATMSCs on Interstitial Fibrosis in Chronic CsA Nephropathy

**Figure 5:**
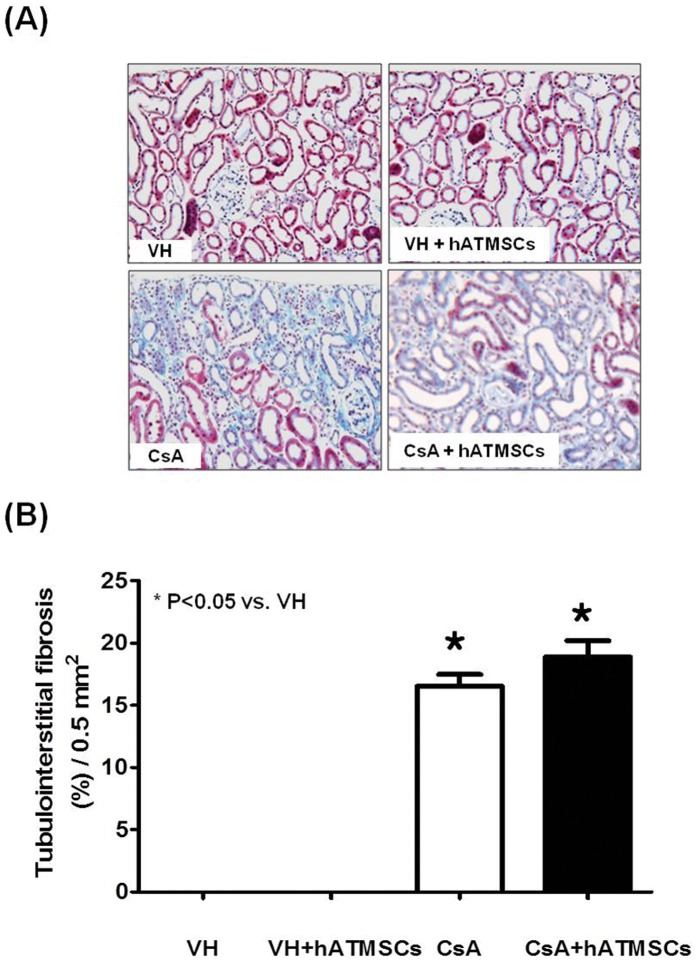
Effect of hATMSCs infusion on interstitial fibrosis in rats with chronic CsA nephropathy. (**A**) Representative photomicrographs of trichrome staining. The CsA group showed typical striped interstitial fibrosis, and extracellular matrix deposition in the cortex (Original magnification x200). (**B**) **Quantitative analysis of tubulointerstitial fibrosis. CsA administration increased tubulointerstitial fibrosis compared to VH and** it was not reduced by treatment with hATMSCs. hATMSCs; human adipose tissue derived mesenchymal stem cells.

Figure 5 shows the results of trichrome staining in the four groups. Typical striped interstitial fibrosis was observed in the kidneys of CsA-treated rats. Quantitative analysis of TIF revealed a significantly higher TIF score in the CsA group than in the VH group (16.5% ±2.5% vs. 0.0%, P<0.05). The TIF score was also higher in the CsA+hATMSC group (18.9% ±3.7%) than in the VH group (P<0.05) but did not differ significantly from that in the CsA group (P>0.05).

### Effects of Treatment with hATMSCs on Apoptotic Cell Death in Chronic CsA Nephropathy

**Figure 6:**
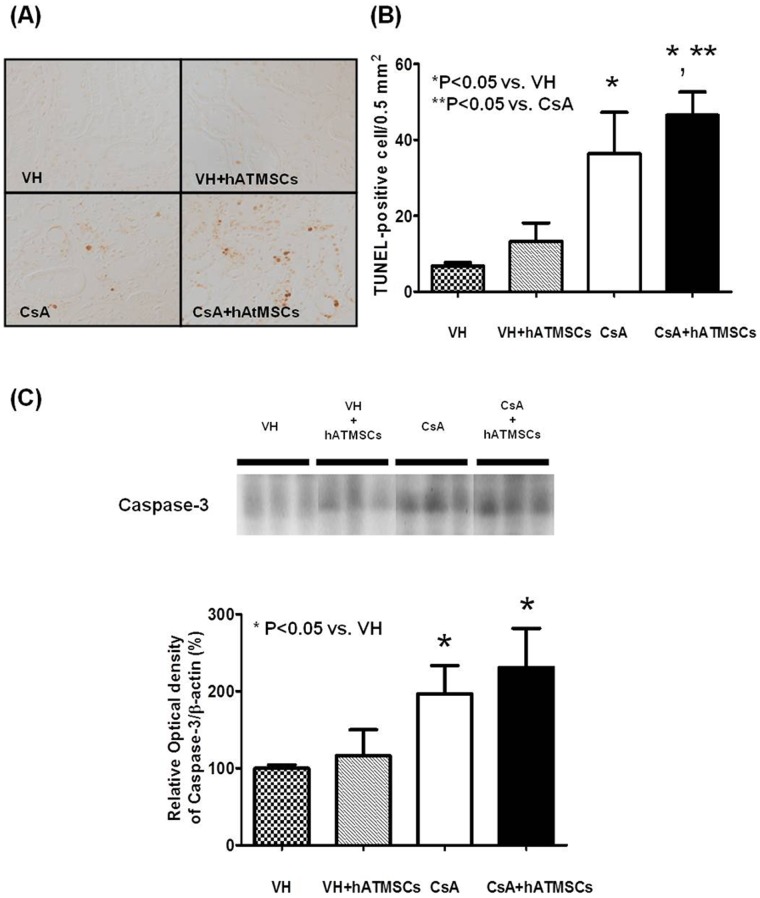
Effect of hATMSCs infusion on apoptosis in rats with chronic CsA nephropathy. (**A**) Representative photomicrographs of TUNEL staining in experimental groups (Original magnification x200). (**B**) Quantitative analysis of TUNEL-positive cells. CsA administration increased TUNEL-positive cell number compared to VH and treatment with hATMSCs further increased it (**C**) Western blot analysis of caspase-3 protein expression. Caspase-3 was activated after treatment with CsA and treatment with hATMSCs did not reduce it. hATMSCs; human adipose tissue derived mesenchymal stem cells.

Figure 6A, B shows TUNEL staining and quantitative analysis in the four groups. A minimal number of TUNEL-positive cells was detected in the VH and VH+hATMSC groups (6.7±1.9/0.5 mm^2^ and 13.3±9.7/0.5 mm^2^, respectively). The number of TUNEL-positive cells was significantly higher in the CsA group (36.3±26.7/0.5 mm^2^) than in the VH group (P<0.05) and in the CsA+hATMSC group (46.5±15.0/0.5 mm^2^) than in the CsA group (P<0.05). Western blot analysis showed that expression of caspase 3 protein was significantly higher in the CsA group (325% ±108%) than in the VH and VH+hATMSC groups (136% ±11.9% and 159% ±24%, respectively; P<0.05). Active caspase 3 protein level did not differ significantly between the CsA+hATMSC group (343% ±172%) and the CsA group (P>0.05) (Figure 6C).

### Effect of hATMSCs on 8-OHdG level in Serum and 24-h Urine in Chronic CsA Nephropathy

**Figure 7:**
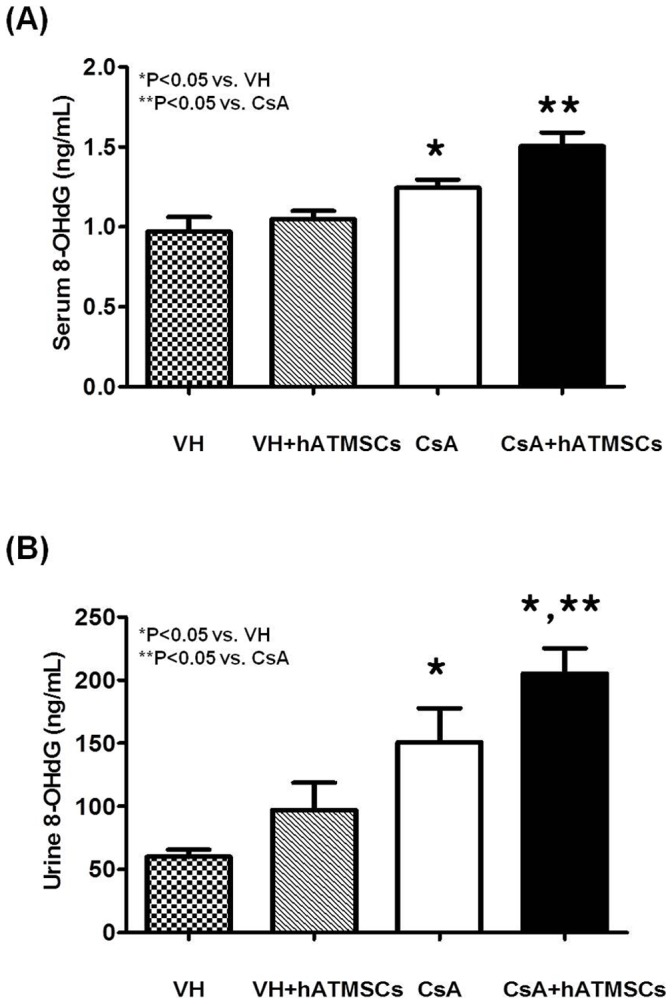
Effect of hATMSCs infusion on oxidative damage in rats with chronic CsA nephropathy. (**A**) The serum and (**B**) urinary concentrations of 8-OHdG was increased in the CsA group, and further increased with the treatment of hATMSCs. hATMSCs; human adipose tissue derived mesenchymal stem cells.

To gain an insight into the mechanisms underlying the lack of protective effects of hATMSCs against chronic CsA nephropathy, serum and urinary 8-OHdG levels were measured as indicators of oxidative stress (Figure 7). Serum 8-OHdG level was significantly higher in the CsA group than in the VH group (0.97±0.09 ng/mL and 1.24±0.05 ng/mL, respectively; P<0.05). Serum 8-OHdG level was significantly higher in the CsA+hATMSC group (1.5±0.09 ng/mL) than in the CsA group (P<0.05). Urinary excretion of 8-OHdG was also significantly higher in the CsA group than in the VH group (150.6±26.9 and 205.1±19.9 ng/mL, respectively; P<0.05). Urinary 8-OHdG excretion was significantly higher in the CsA+hATMSC group than in the CsA group (205.1±19.9 ng/dL, P<0.05).

### Apoptosis of HK-2 Cells Cultured in hATMSC-conditioned Media with and without CsA

**Figure 8:**
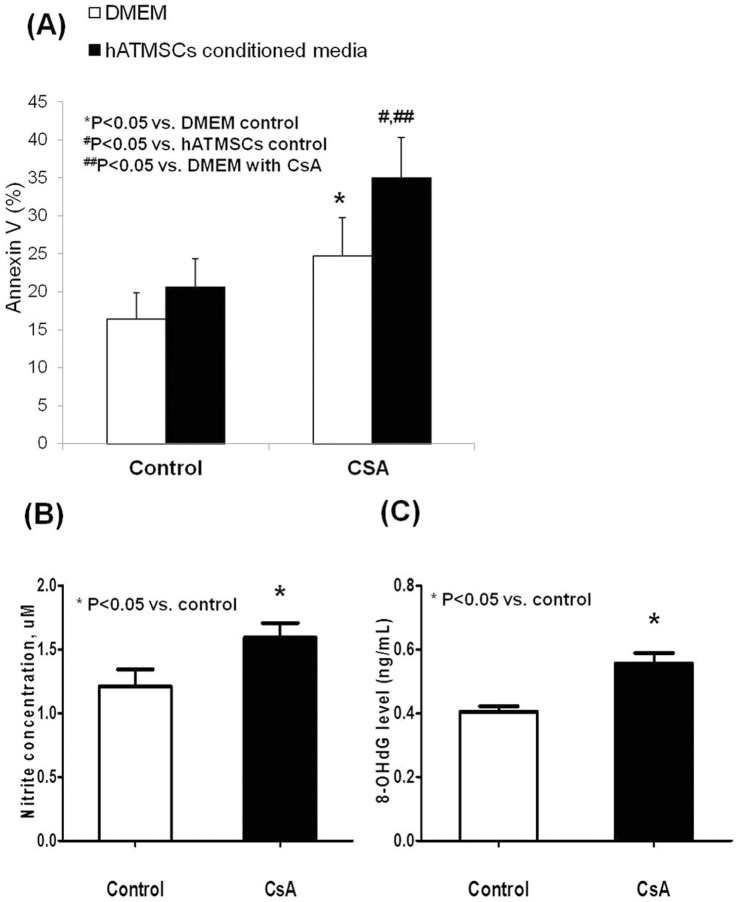
The results of in vitro study. (**A**) The number of Annexin V positive cell didn’t increase in HK-2 cell cultured in hATMSCs- conditioned medium compared to HK-2 cell cultured in DMEM. Addition of CsA increased Annexin V positive cell both in DMED and hATMSCs-conditioned medium, and it was more frequently detected in HK-2 cell cultured in hATMSCs-conditioned medium compared to HK-2 cells cultured in DMEM. (**B**) The production of NO^−^ was increased in medium from hATMSCs treated with CsA compared with medium from hATMSCs cultured without CsA. (**C**) The 8-OHdG level was significantly increased in the medium from hATMSCs treated with CsA compared to medium from hATMSCs cultured withCsA-free media as well. hATMSCs; human adipose tissue derived mesenchymal stem cells.

To investigate the effect of CsA and hATMSC-conditioned medium on apoptosis of HK-2 cells, Annexin V-positive HK-2 cells were detected using flow cytometry. The frequency of Annexin V-positive HK-2 cells did not differ between cells cultured in hATMSC-conditioned medium compared with those cultured in DMEM. Addition of CsA increased the frequency of Annexin V-positive cells exposed to DMEM and hATMSC-conditioned media. However, Annexin V-positive cells were more frequent in HK-2 cells cultured in hATMSC-conditioned medium than in those cultured in DMEM (P<0.05) (Figure 8A).

### Oxidative Stress Markers in hATMSC-conditioned Media with and without CsA

To evaluate the effect of CsA on oxidative stress markers produced by hATMSCs, we compared NO^–^ and 8-OHdG levels in hATMSC-conditioned media from cells incubated with and without CsA at a concentration of 100 µM. The NO^–^ level was significantly higher in medium from hATMSCs cultured with CsA than in medium from cells cultured without CsA (1.6±0.1 µM and 1.2±0.1 µM, respectively; P<0.05) (Figure 8B). The 8-OHdG level was significantly higher in medium from hATMSCs incubated with CsA than in medium from cells incubated without CsA (0.56±0.06 ng/mL and 0.40±0.03 ng/mL, respectively; P<0.05) (Figure 8C).

## Discussion

We planned this study to evaluate the beneficial effect of hATMSCs on CsA-iduced renal injury. But the results of our study show that hATMSC treatment is not effective in reducing or preventing renal injury in an experimental model of chronic CsA nephropathy. In addition, renal tissue injury indicators such as apoptotic cell death and interstitial fibrosis were aggravated in the CsA+hATMSC group compared with the CsA monotherapy group. Therefore, we studied the mechanism responsible for the deleterious effect of hATMSC treatment on CsA-induced renal injury and found that increased oxidative stress caused by combined treatment with CsA and hATMSCs was responsible. The results of our study suggest that hATMSCs can be a source of oxidative stress in pathological conditions such as chronic CsA nephropathy. This may explain the adverse effects of MSCs in some previous reports [17], [18], [19], [20].

First, we investigated whether systemically infused hATMSCs migrate to the kidney, and we compared the cell numbers between the CsA and VH groups. hATMSCs were detected in tissues from both groups, but more hATMSCs were detected in the CsA group. MSCs preferentially migrate to or dock in injured sites when infused in animal models of injury; this property can be attributed to the expression of growth factors, chemokines, and extracellular matrix receptors on the surface of MSCs [21], [22], [23]. It is possible that CsA-induced renal injury upregulates several molecules whose presence causes retention of systemically infused MSCs. One study showed recruitment of nestin-expressing cells to areas of CsA-induced renal injury where they play a role in the repair of injured tissues and organs [24].

Second, we investigated the effect of hATMSCs on CsA-induced renal injury. Despite successful migration of hATMSCs to injured renal tissue in the CsA group, this did not protect against CsA-induced renal tissue injury. Moreover, apoptotic cell death and inflammatory cell infiltration were significantly higher in the CsA+hATMSC group than in the CsA group. Consequently, renal function and TIF showed a deteriorating tendency in the CsA+hATMSC group. The in vitro study showed greater CsA-induced apoptosis in HK-2 cells cultured in hATMSC-conditioned medium, which is consistent with the results of our in vivo study. By contrast, renal injury indicators did not differ significantly between the VH+hATMSC group and the VH group. These findings suggest that the combination of CsA and hATMSCs aggravates renal injury beyond that caused by CsA alone.

Our next consideration was the mechanism through which hATMSCs aggravate CsA-induced renal injury. The levels of oxidative stress markers were higher in both serum and urine in the CsA+hATMSC group compared with the CsA group. Oxidative stress is an important mechanism in the progression of CsA-induced renal injury, and oxidative stress markers such as 8-OHdG are regarded as key mediators of CsA-induced renal tissue injury [25], [26], [27]. Our results suggest that the increased oxidative stress in the CsA+hATMSC group mediated the aggravation of renal tissue injury in this group.

With regard to the reason of the increase of 8-OHdG, we hypothesized that high concentration of CsA may be toxic to infused hATMSCs, hence it may affect hATMSCs to secret oxidative stress marker. To prove it, we performed the in vitro study of the effect of CsA on the production of oxidative stress markers by hATMSCs. The results showed higher levels of NO^–^ and 8-OHdG in hATMSC-conditioned culture medium from cells exposed to CsA compared with medium from cells cultured without CsA. This suggests that CsA triggers hATMSCs to secrete oxidative stress markers, which may be associated with the systemic increase of oxidative stress. Another possible reason is that combined CsA and hATMSCs therapy may act in different organs such as liver or heart, which could be damaged by CsA [28], [29]. It may result in increased serum oxidative stress markers. But further investigations may be required for clear conclusion about it.

Another important reason for the lack of protection by hATMSCs is that the therapeutic effect of hATMSCs is influenced by environmental factors. hATMSCs need to be “licensed” in an appropriate cytokine environment before they can exert their actions [30]. It is possible that CsA disrupts this essential environmental condition and that the function of hATMSCs is consequently suppressed. For example, MSCs significantly suppress alloreactivity in both in vitro and in vivo transplant models [31], [32], [33]. However, CsA given with MSCs did not prolong animal and allograft survival and even accelerated acute allograft rejection in an organ transplant rat model [17], [18]. It has been proposed that calcineurin inhibitor antagonizes the effect of MSCs by inhibiting the proinflammatory microenvironment required for their activation [17], [34].

The characteristics of animal models may also affect the microenvironment of hATMSCs. In our chronic CsA nephropathy model, CsA was injected daily, and the blood and tissue CsA levels remained high during the experimental period. Therefore, the infused hATMSCs would have been exposed to a microenvironment of high CsA concentration, which may have inhibited their functions for the reason described above. The 8-OHdG level increased in both urine and serum, suggesting a systemic effect of CsA on the infused hATMSCs. By contrast, in other kidney disease models in which MSCs showed beneficial effects, the MSC microenvironment did not change during the experiments because inflammation was confined mainly to the kidney and MSCs were not influenced directly by the source of renal injury such as ischemic–reperfusion or nephrotoxic drugs [35], [36], [37], [38].

Our study does not suggest that hATMSCs themselves have detrimental effects on renal tissue. In this study, hATMSC treatment alone (without CsA) did not have any harmful effects in terms of renal injury indicators or renal function, and hATMSC treatment alone did not increase oxidative stress compared with VH. In addition, previous studies have shown that the antioxidative stress effect is an important mechanism of MSCs in the protection of organ injury [39], [40], [41], [42]. Our data suggest only that the tissue-repairing function of hATMSCs may be deficient when they are administered in an environment containing high concentrations of CsA.

One question is whether the xenogenicity of hATMSCs may be the cause of the lack of protective effect by hATMSCs in animal models. However, the safety and effectiveness of hATMSCs have been established in previous studies using experimental animal models [14], [37], [43]. Moreover, we used human renal tubular cell lines in the in vitro study to perform it to exclude the effect of xenogenicity in this study. Nevertheless, apoptosis of human HK-2 cells significantly increased under both CsA and hATMSCsand NO^–^ and 8-OHdG levels were higher in hATMSC-conditioned medium from cells exposed to CsA, similar to the results of our animal study. This suggests that the xenogenicity may not be responsible for the increased oxidative stress and the loss of protective effect of hATMSCs in this study. In addition, because cells of human origin will be used in future clinical trials, we thought that their efficacy should be assessed in an animal setting before their use in clinical trials.

In conclusion, despite promising results for the use of MSCs in kidney disease models, it is important to consider the environmental factors that are likely to significantly affect the biological properties of MSCs. Our results suggest that hATMSCs play different action according to the different model and environment.Therefore, we cautiously conclude that the beneficial function of hATMSCs is diminished by environment with high concentrations of CsA.

## References

[1] Bennett WM, DeMattos A, Meyer MM, Andoh T, Barry JM (1996) Chronic cyclosporine nephropathy: the Achilles’ heel of immunosuppressive therapy. Kidney Int 50: 1089–1100. 8887265 10.1038/ki.1996.415

[2] Myers BD, Ross J, Newton L, Luetscher J, Perlroth M (1984) Cyclosporine-associated chronic nephropathy. N Engl J Med 311: 699–705. 6382005 10.1056/NEJM198409133111103

[3] Wang C, Salahudeen AK (1995) Lipid peroxidation accompanies cyclosporine nephrotoxicity: effects of vitamin E. Kidney Int. 47: 927–934. 10.1038/ki.1995.138 7752594

[4] Yang CW, Faulkner GR, Wahba IM, Christianson TA, Bagby GC, et al (2002) Expression of apoptosis-related genes in chronic cyclosporine nephrotoxicity in mice. Am J Transplant 2: 391–399. 12123203 10.1034/j.1600-6143.2002.20501.x

[5] Yoon HE, Yang CW (2009) Established and newly proposed mechanisms of chronic cyclosporine nephropathy. Korean J Intern Med 24: 81–92. 19543484 10.3904/kjim.2009.24.2.81 PMC2698583

[6] Picinich SC, Mishra PJ, Glod J, Banerjee D (2007) The therapeutic potential of mesenchymal stem cells. Cell- & tissue-based therapy. Expert Opin Biol Ther 7: 965–973. 17665987 10.1517/14712598.7.7.965

[7] Nauta AJ, Fibbe WE (2007) Immunomodulatory properties of mesenchymal stromal cells. Blood 110: 3499–3506. 17664353 10.1182/blood-2007-02-069716

[8] Hematti P (2008) Role of mesenchymal stromal cells in solid organ transplantation. Transplant Rev (Orlando) 22: 262–273. 18656340 10.1016/j.trre.2008.05.002 PMC2576746

[9] Meirelles Lda S, Fontes AM, Covas DT, Caplan AI (2009) Mechanisms involved in the therapeutic properties of mesenchymal stem cells. Cytokine Growth Factor Rev 20: 419–427. 19926330 10.1016/j.cytogfr.2009.10.002

[10] Li B, Cohen A, Hudson TE, Motlagh D, Amrani DL, et al (2010) Mobilized human hematopoietic stem/progenitor cells promote kidney repair after ischemia/reperfusion injury. Circulation 121: 2211–2220. 20458011 10.1161/CIRCULATIONAHA.109.928796 PMC2919223

[11] Togel F, Weiss K, Yang Y, Hu Z, Zhang P, et al (2007) Vasculotropic, paracrine actions of infused mesenchymal stem cells are important to the recovery from acute kidney injury. Am J Physiol Renal Physiol 292: F1626–1635. 17213465 10.1152/ajprenal.00339.2006

[12] Bi B, Schmitt R, Israilova M, Nishio H, Cantley LG (2007) Stromal cells protect against acute tubular injury via an endocrine effect. J Am Soc Nephrol 18: 2486–2496. 17656474 10.1681/ASN.2007020140

[13] Dekel B, Shezen E, Even-Tov-Friedman S, Katchman H, Margalit R, et al (2006) Transplantation of human hematopoietic stem cells into ischemic and growing kidneys suggests a role in vasculogenesis but not tubulogenesis. Stem Cells 24: 1185–1193. 16410390 10.1634/stemcells.2005-0265

[14] Ra JC, Shin IS, Kim SH, Kang SK, Kang BC, et al (2011) Safety of intravenous infusion of human adipose tissue-derived mesenchymal stem cells in animals and humans. Stem Cells Dev 20: 1297–1308. 21303266 10.1089/scd.2010.0466

[15] Sagrinati C, Netti GS, Mazzinghi B, Lazzeri E, Liotta F, et al (2006) Isolation and characterization of multipotent progenitor cells from the Bowman’s capsule of adult human kidneys. J Am Soc Nephrol 17: 2443–2456. 16885410 10.1681/ASN.2006010089

[16] Ghee JY, Han DH, Song HK, Kim WY, Kim SH, et al (2008) The role of macrophage in the pathogenesis of chronic cyclosporine-induced nephropathy. Nephrol Dial Transplant 23: 4061–4069. 18622021 10.1093/ndt/gfn388

[17] Hoogduijn MJ, Crop MJ, Korevaar SS, Peeters AM, Eijken M, et al (2008) Susceptibility of human mesenchymal stem cells to tacrolimus, mycophenolic acid, and rapamycin. Transplantation 86: 1283–1291. 19005411 10.1097/TP.0b013e31818aa536

[18] Zhang W, Qin C, Zhou ZM (2007) Mesenchymal stem cells modulate immune responses combined with cyclosporine in a rat renal transplantation model. Transplant Proc 39: 3404–3408. 18089393 10.1016/j.transproceed.2007.06.092

[19] Horn AP, Bernardi A, Luiz Frozza R, Grudzinski PB, Hoppe JB, et al (2011) Mesenchymal stem cell-conditioned medium triggers neuroinflammation and reactive species generation in organotypic cultures of rat hippocampus. Stem cells and development 20: 1171–1181. 20955077 10.1089/scd.2010.0157

[20] Horn AP, Frozza RL, Grudzinski PB, Gerhardt D, Hoppe JB, et al (2009) Conditioned medium from mesenchymal stem cells induces cell death in organotypic cultures of rat hippocampus and aggravates lesion in a model of oxygen and glucose deprivation. Neuroscience research 63: 35–41. 18977399 10.1016/j.neures.2008.10.001

[21] da Silva Meirelles L, Caplan AI, Nardi NB (2008) In search of the in vivo identity of mesenchymal stem cells. Stem Cells 26: 2287–2299. 18566331 10.1634/stemcells.2007-1122

[22] Rojas M, Xu J, Woods CR, Mora AL, Spears W, et al (2005) Bone marrow-derived mesenchymal stem cells in repair of the injured lung. Am J Respir Cell Mol Biol 33: 145–152. 15891110 10.1165/rcmb.2004-0330OC PMC2715309

[23] Ponte AL, Marais E, Gallay N, Langonne A, Delorme B, et al (2007) The in vitro migration capacity of human bone marrow mesenchymal stem cells: comparison of chemokine and growth factor chemotactic activities. Stem Cells 25: 1737–1745. 17395768 10.1634/stemcells.2007-0054

[24] Ahn KO, Li C, Lim SW, Song HK, Ghee JY, et al (2008) Infiltration of nestin-expressing cells in interstitial fibrosis in chronic cyclosporine nephropathy. Transplantation 86: 571–577. 18724228 10.1097/TP.0b013e3181820470

[25] Redondo-Horcajo M, Lamas S (2005) Oxidative and nitrosative stress in kidney disease: a case for cyclosporine A. J Nephrol. 18: 453–457. 16245254

[26] Yoon HE, Ghee JY, Piao S, Song JH, Han DH, et al (2011) Angiotensin II blockade upregulates the expression of Klotho, the anti-ageing gene, in an experimental model of chronic cyclosporine nephropathy. Nephrol Dial Transplant 26: 800–813. 20813770 10.1093/ndt/gfq537 PMC3108350

[27] Han DH, Piao SG, Song JH, Ghee JY, Hwang HS, et al (2010) Effect of sirolimus on calcineurin inhibitor-induced nephrotoxicity using renal expression of KLOTHO, an antiaging gene. Transplantation 90: 135–141. 20562737 10.1097/TP.0b013e3181e117b4

[28] Deters M, Strubelt O, Younes M (1997) Reevaluation of cyclosporine induced hepatotoxicity in the isolated perfused rat liver. Toxicology 123: 197–206. 9355938 10.1016/s0300-483x(97)00123-6

[29] Owunwanne A, Shihab-Eldeen A, Sadek S, Junaid T, Yacoub T, et al (1993) Is cyclosporine toxic to the heart? J Heart Lung Transplant 12: 199–204. 8476891

[30] Djouad F, Fritz V, Apparailly F, Louis Plence P, Bony C, et al (2005) Reversal of the immunosuppressive properties of mesenchymal stem cells by tumor necrosis factor alpha in collagen-induced arthritis. Arthritis 52: 1595–1603. 10.1002/art.21012 15880818

[31] Crop MJ, Baan CC, Korevaar SS, Ijzermans JN, Alwayn IP, et al (2009) Donor-derived mesenchymal stem cells suppress alloreactivity of kidney transplant patients. Transplantation 87: 896–906. 19300194 10.1097/TP.0b013e31819b3d72

[32] Muller MH, Poncet C, Prosperi JM, Santoni S, Ronfort J (2006) Domestication history in the Medicago sativa species complex: inferences from nuclear sequence polymorphism. Mol Ecol 15: 1589–1602. 16629813 10.1111/j.1365-294X.2006.02851.x

[33] Zhou HP, Yi DH, Yu SQ, Sun GC, Cui Q, et al (2006) Administration of donor-derived mesenchymal stem cells can prolong the survival of rat cardiac allograft. Transplant Proc 38: 3046–3051. 17112896 10.1016/j.transproceed.2006.10.002

[34] Inoue S, Popp FC, Koehl GE, Piso P, Schlitt HJ, et al (2006) Immunomodulatory effects of mesenchymal stem cells in a rat organ transplant model. Transplantation 81: 1589–1595. 16770249 10.1097/01.tp.0000209919.90630.7b

[35] Semedo P, Correa-Costa M, Antonio Cenedeze M, Maria Avancini Costa Malheiros D, Antonia dos Reis M, et al (2009) Mesenchymal stem cells attenuate renal fibrosis through immune modulation and remodeling properties in a rat remnant kidney model. Stem Cells 27: 3063–3073. 19750536 10.1002/stem.214

[36] Togel F, Hu Z, Weiss K, Isaac J, Lange C, et al (2005) Administered mesenchymal stem cells protect against ischemic acute renal failure through differentiation-independent mechanisms. Am J Physiol Renal Physiol 289: F31–42. 15713913 10.1152/ajprenal.00007.2005

[37] Kim JH, Park DJ, Yun JC, Jung MH, Yeo HD, et al (2012) Human adipose tissue-derived mesenchymal stem cells protect kidneys from cisplatin nephrotoxicity in rats. Am J Physiol Renal Physiol 302: F1141–1150. 22205231 10.1152/ajprenal.00060.2011

[38] Magnasco A, Corselli M, Bertelli R, Ibatici A, Peresi M, et al (2008) Mesenchymal stem cells protective effect in adriamycin model of nephropathy. Cell Transplant 17: 1157–1167. 19181210 10.3727/096368908787236567

[39] Mohammadzadeh M, Halabian R, Gharehbaghian A, Amirizadeh N, Jahanian-Najafabadi A, et al (2012) Nrf-2 overexpression in mesenchymal stem cells reduces oxidative stress-induced apoptosis and cytotoxicity. Cell Stress Chaperones 17: 553–565. 22362068 10.1007/s12192-012-0331-9 PMC3535169

[40] Sun CK, Chang CL, Lin YC, Kao YH, Chang LT, et al (2012) Systemic administration of autologous adipose-derived mesenchymal stem cells alleviates hepatic ischemia-reperfusion injury in rats. Crit Care Med 40: 1279–1290. 22336724 10.1097/CCM.0b013e31823dae23

[41] Whone AL, Kemp K, Sun M, Wilkins A, Scolding NJ (2012) Human bone marrow mesenchymal stem cells protect catecholaminergic and serotonergic neuronal perikarya and transporter function from oxidative stress by the secretion of glial-derived neurotrophic factor. Brain Res 1431: 86–96. 22143094 10.1016/j.brainres.2011.10.038

[42] Chen YT, Sun CK, Lin YC, Chang LT, Chen YL, et al (2011) Adipose-derived mesenchymal stem cell protects kidneys against ischemia-reperfusion injury through suppressing oxidative stress and inflammatory reaction. J Transl Med 9: 51. 21545725 10.1186/1479-5876-9-51 PMC3112438

[43] Choi HJ, Kim JM, Kwon E, Che JH, Lee JI, et al (2011) Establishment of efficacy and safety assessment of human adipose tissue-derived mesenchymal stem cells (hATMSCs) in a nude rat femoral segmental defect model. J Korean Med Sci 26: 482–491. 21468254 10.3346/jkms.2011.26.4.482 PMC3069566

